# Cavitation Dynamics and Inertial Cavitation Threshold of Lipid Coated Microbubbles in Viscoelastic Media with Bubble–Bubble Interactions

**DOI:** 10.3390/mi12091125

**Published:** 2021-09-18

**Authors:** Dui Qin, Qingqin Zou, Shuang Lei, Wei Wang, Zhangyong Li

**Affiliations:** Chongqing Engineering Research Center of Medical Electronics and Information Technology, Department of Biomedical Engineering, School of Bioinformatics, Chongqing University of Posts and Telecommunications, Chongqing 400065, China; s200502007@stu.cqupt.edu.cn (Q.Z.); ls819055128@126.com (S.L.); wangw@cqupt.edu.cn (W.W.)

**Keywords:** ultrasound, lipid-coated microbubble, inertial cavitation, viscoelasticity, bubble–bubble interaction

## Abstract

Encapsulated microbubbles combined with ultrasound have been widely utilized in various biomedical applications; however, the bubble dynamics in viscoelastic medium have not been completely understood. It involves complex interactions of coated microbubbles with ultrasound, nearby microbubbles and surrounding medium. Here, a comprehensive model capable of simulating the complex bubble dynamics was developed via taking the nonlinear viscoelastic behaviors of the shells, the bubble–bubble interactions and the viscoelasticity of the surrounding medium into account simultaneously. For two interacting lipid-coated bubbles with different initial radii in viscoelastic media, it exemplified that the encapsulating shell, the inter-bubble interactions and the medium viscoelasticity would noticeably suppress bubble oscillations. The inter-bubble interactions exerted a much stronger suppressing effect on the small bubble within the parameters examined in this paper, which might result from a larger radiated pressure acting on the small bubble due to the inter-bubble interactions. The lipid shells make the microbubbles exhibit two typical asymmetric dynamic behaviors (i.e., compression or expansion dominated oscillations), which are determined by the initial surface tension of the bubbles. Accordingly, the inertial cavitation threshold decreases as the initial surface tension increases, but increases as the shell elasticity and viscosity increases. Moreover, with the distance between bubbles decreasing and/or the initial radius of the large bubble increasing, the oscillations of the small bubble decrease and the inertial cavitation threshold increases gradually due to the stronger suppression effects caused by the enhanced bubble–bubble interactions. Additionally, increasing the elasticity and/or viscosity of the surrounding medium would also dampen bubble oscillations and result in a significant increase in the inertial cavitation threshold. This study may contribute to both encapsulated microbubble-associated ultrasound diagnostic and emerging therapeutic applications.

## 1. Introduction

Encapsulated microbubbles in combination with ultrasound have gained much attention for various biomedical applications [1,2,3,4,5,6,7]. They are usually composed of a low solubility gas core and a stabilizing shell made up of lipids, denatured albumins or polymers. The microbubbles usually have sizes between 1 and 10 microns, making them suitable for intravenous injection [3,4,8,9]. These microbubbles are originally utilized as contrast agents in clinic for enhancing ultrasound diagnostic imaging owing to their higher echogenicity than those of the tissues [1,4]. Additionally, the encapsulated microbubbles have recently shown growing potential in therapeutic ultrasound applications, such as sonothrombolysis, targeted drug/gene delivery, transient opening of the blood−brain barrier, enhancement of high intensity focused ultrasound (HIFU) treatment and so on [2,3,4,5,6,7,10,11]. When subjected to ultrasound irradiation, the microbubbles would undergo complex and transient dynamic behaviors (i.e., growth, oscillation and collapse), termed as acoustic cavitation. Consequently, the ultrasound-induced bioeffects or therapeutic outcomes can be significantly enhanced due to the cavitation of microbubbles [1,2,3,4,5,6,7,8]. According to the dynamic behaviors of microbubbles, the acoustic cavitation can be classified into stable cavitation, oscillating about a certain equilibrium size with a small amplitude and a lifetime of many cycles, or inertial cavitation in which microbubbles oscillate with a large amplitude followed by a rapid violent collapse [12,13]. For different medical applications, the microbubble cavitation could be either therapeutically beneficial or undesirable, which depends on the cavitation type and intensity. Increasing the ultrasonic pressure above a certain threshold could result in the transition from stable cavitation to inertial cavitation. To determine the onset of inertial cavitation, some criteria have been established, such as *R*_max_/*R*_0_ = 2 [14,15,16,17,18], *R*_max_/*R*_0_ = 2.3 [19], *R*_max_/*R*_min_ = 10 [20] and so on, where *R*_max_ and *R*_min_ are the maximum and minimum bubble radii, respectively; *R*_0_ is the initial bubble radius. In this study, it may be reasonable to choose *R*_max_/*R*_0_ = 2 as the criterion of inertial cavitation for safety considerations, since it seems to be the minimum threshold of bubble destruction as observed experimentally [21,22,23]. Therefore, it is of considerable importance to investigate the cavitation dynamics of microbubbles in biological tissues and determine the threshold pressure of inertial cavitation for optimal utilization of microbubbles.

To understand the complex cavitation dynamics of encapsulated microbubbles, several numerical investigations with most attempts to modify the classic equation of cavitation dynamics have been performed by considering the effects of shell properties, such as surface tension and dilatational viscosity [24,25,26,27,28,29,30,31,32,33,34,35,36,37,38,39,40]. Among various encapsulating shells, the lipid shell is more flexible, which can make the microbubbles more prone to occurring cavitation under low-amplitude ultrasound irradiation and it is highly attractive for cavitation-assisted therapeutic applications. Moreover, the lipid-coated microbubbles deserve more attention because of their current prevalence among clinical agents. Considering the complex interfacial rheology of the lipid shell, some numerical models were further proposed to reasonably simulate the typical asymmetric “compression-only” behavior of the lipid-coated microbubbles [24,26,27,29,32,35,36,37,38,39,40]. Marmottant et al. modeled the asymmetric large-amplitude oscillations of lipid-coated microbubbles by taking the buckling and rupture of the lipid shell into account, whereby the effective surface tension of the lipid shell was separated along three different regimes during the bubble expansion and compression [24]. For modeling the shear-thinning behavior of the lipid-coated microbubbles, Doinikov et al. modified the Rayleigh–Plesset (RP) equation by introducing the Cross law to model the nonlinear shell viscosity as a function of the shell shear rate, while keeping the shell elastic modulus constant [40]. Tu et al. used three different models (i.e., Hoff’s, Sarkar’s and linearized Marmottant’s models) to analyze the radial behavior of SonoVue and then compared the results with experimental outputs, demonstrating that these models have similar results at linear vibrations [26]. Furthermore, by applying the Cross law to the nonlinear shell viscous term in the Marmottant model, Li et al. proposed a new model with considering the nonlinear changes of both shell viscosity and elasticity simultaneously [32]. Compared with the previous models, this model can reduce the dependence of bubble shell parameters on the initial bubble radius.

Moreover, in the aforementioned ultrasound applications combined with the encapsulated microbubbles, the medium surrounding the microbubbles is blood or soft tissue that displays certain viscoelasticity. Therefore, it is essential to consider the viscoelastic features of the surrounding medium when investigating the microbubble dynamics and the inertial cavitation threshold. Until now, various viscoelastic models for describing the viscoelastic medium exist (e.g., Kelvin-Voigt, Maxwell, Zener, etc.) and several of them have been coupled with the cavitation models (e.g., RP equation, Keller-Miksis equation, Gilmore equation, etc.) to investigate the dynamic behaviors of a single microbubble in viscoelastic fluids or tissues for a sake of simplicity [15,28,34,41,42]. However, multiple bubbles or even a bubble cloud are generally present in the majority of cavitation-assisted ultrasound applications, therefore the influence of inter-bubble interactions should not be ignored. Specifically, secondary radiation forces are generated between oscillating microbubbles, causing the cavitation dynamics and the inertial cavitation threshold of interacting microbubbles being extremely different from those of an individual microbubble. For the bubble–bubble interactions, much research has focused on the translational motions or radial pulsations of bubbles in Newtonian fluids [43,44,45,46,47,48,49,50,51,52,53,54]. It has been demonstrated that the expansion ratios of bubbles can be suppressed or enlarged, mainly depending on the ultrasound parameters, the initial bubble radii, the bubble–bubble distance and the number of bubbles [43,44,45,46,47,48,49,50,51,52,53,54]. Recently, a few studies have begun to pay attention to the translational and/or radial motions of uncoated microbubbles in viscoelastic medium with considering the bubble–bubble interactions [55,56,57]. It suggests that the translational and radial motions of bubbles could be reduced significantly with the medium elasticity, viscosity or both of them increasing, leading to a decrease in the bubble–bubble interactions [55,57]. Therefore, in ultrasound applications with encapsulated microbubbles, the microbubble dynamics and the inertial cavitation threshold are strongly influenced by the ultrasound parameters, the shell properties, the viscoelasticity of the surrounding medium and the bubble–bubble interactions, which need to be comprehensively investigated for controlling cavitation activity to harness its biomedical potentials.

In this study, a comprehensive model was developed to investigate the dynamic behaviors and the inertial cavitation threshold of lipid-coated microbubbles in viscoelastic tissues, with simultaneously considering the viscoelastic properties of the shell and the surrounding medium as well as the bubble–bubble interactions. The dependence of the radial oscillations (or expansion ratio) and the inertial cavitation threshold of microbubbles on the shell properties, the bubble–bubble interactions and the viscoelasticity of the surrounding medium was presented, respectively. Understanding these complex dependencies may offer scalable strategies for properly controlling the bubble dynamics and cavitation type/activity, which may be greatly valuable for selectively enhancing the diagnostic and/or therapeutic ultrasound applications associated with lipid-coated microbubbles.

## 2. Theory and Methods

### 2.1. Modelling the Cavitation Dynamics of Encapsulated Microbubbles in Viscoelastic Media with Considering Bubble–Bubble Interactions

**Figure 1:**
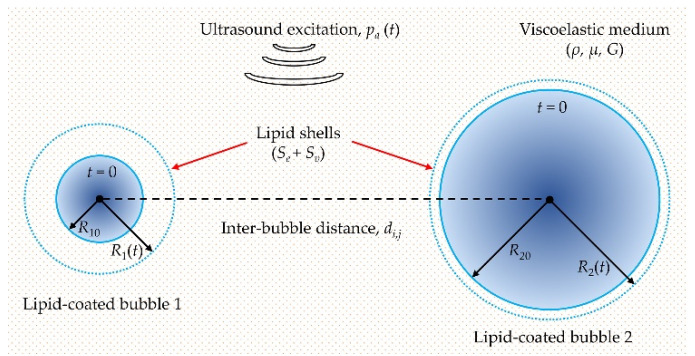
Schematic of the cavitation dynamics for two interacting lipid-coated microbubbles in viscoelastic tissues.

The schematic diagram for describing the dynamic behaviors of two interacting encapsulated microbubbles in a viscoelastic medium is shown in Figure 1. Under ultrasound excitation, two microbubbles with initial radii (*R*_10_ and *R*_20_) could occur radial oscillations over time, i.e., *R*_1_(*t*) and *R*_2_(*t*). It is assumed that both microbubbles remain spherical during the oscillations, and the mass exchange at the gas–liquid interfaces, the chemical reactions inside the microbubbles as well as the translational motions of the microbubbles are neglected as described in earlier studies [44,46,48,50,53,54]. The Keller-Miksis equations coupled with the bubble–bubble interactions were used as follows [48,53]:

(1) $$\left(1-\frac{{\dot{R}}_{i}}{c}\right)R_{i}{\ddot{R}}_{i}+\left(\frac{3}{2}-\frac{{\dot{R}}_{i}}{2c}\right){\dot{R}}_{i}^{2}=\frac{1}{\rho }\left(1+\frac{{\dot{R}}_{i}}{c}\right)p_{s,i}+\frac{R_{i}{\dot{p}}_{s,i}}{\rho c}-\sum_{j=1,j\neq i}^{2}\frac{\left(2R_{j}{\dot{R}}_{j}^{2}+R_{j}^{2}{\ddot{R}}_{j}\right)}{d_{i,j}}$$

where *R_i_*(*t*), *R_j_*(*t*) are the time-varying radii of the *i*th and *j*th microbubble, respectively. The indexes *i* = 1, 2 and *j* = 3 − *i* denote the bubble number. The overdot denotes the time derivative. The *c* is the speed of sound in the surrounding medium, *ρ* is the medium density, *d_i,j_* is the distance between the centers of the *i*th and *j*th microbubbles, *p_s,i_*(*t*) is the pressure in the surrounding medium at the wall of *i*th microbubble. Note that the time delay while the pressure radiated by one bubble propagating to the other bubble was neglected in Equation (1); it is reasonable because the initial bubble−bubble distances are small (*d_i,j_* < 200 μm) in this study, in which case the time delay becomes insignificant. Sojahrood et al. provided a review of the time delays in the simulation of interacting bubbles [54].

### 2.2. Viscoelastic Model for the Surrounding Medium

To describe the viscoelastic behaviors of the surrounding medium, the Zener model was used due to its superiority for describing the relaxation and elasticity behavior of soft tissues at the same time, which was expressed as [41,42,55]:

(2) $$\tau_{rr}+\lambda_{1}{\dot{\tau }}_{rr}=2G\gamma_{rr}+2\mu {\dot{\gamma }}_{rr}$$

where $\tau_{rr}$ is the stress in the *r* direction, $\gamma_{rr}$ is the strain, ${\dot{\gamma }}_{rr}$ is the strain rate, *G*, *μ* and *λ*_1_ are the elasticity modulus, viscosity and relaxation time of the surrounding medium, respectively. According to the continuity equation, one can obtain ${\dot{\gamma }}_{rr,i}=-2R_{i}^{2}{\dot{R}}_{i}/r^{3}$ and $\gamma_{rr,i}=-2\left(R_{i}^{3}-R_{i0}^{3}\right)/3r^{3}$ for the *i*th microbubble, then substituting these conditions into Equation (2), the stress at the interface of the *i*th microbubble (*r* = *R_i_*) was given by:

(3) $$\tau_{rr}|{}_{R_{i}}+{\lambda_{1}{\dot{\tau }}_{rr}|}_{R_{i}}=-\frac{4G}{3}\left(1-\frac{R_{i0}^{3}}{R_{i}^{3}}\right)-4\mu \frac{{\dot{R}}_{i}}{R_{i}}$$

where $\tau_{rr}{|}_{R_{i}}$ is the stress at *r* = *R_i_*, *R_i_*_0_ is the initial radius of the *i*th microbubble.

### 2.3. Modeling the Shell Properties of the Lipid-Coated Microbubbles

To describe large-amplitude oscillations of microbubbles coated with monolayer lipid shells, the nonlinear shell elasticity *S_e_* and shell viscosity *S_v_* were simultaneously considered in our simulations [32]:

(4) $$S=S_{e}+S_{v}=\frac{2\sigma \left(R\right)}{R}+4\kappa_{s}\frac{\dot{R}}{R^{2}}$$

where *κ_s_* is the shell viscosity, *σ*(*R*) is the effective surface tension at radius *R*, which is determined by the Marmottant model [24]:

(5) $$\sigma (R)=\{\begin{array}{c} 0\\ \chi \left(R^{2}/R_{\mathrm{buckling}}^{2}-1\right)\\ \sigma_{\mathrm{tissue}} \end{array}\begin{array}{cc} & \mathrm{if}\;R\leq R_{\mathrm{buckling}}\\ & \mathrm{if}\;R_{\mathrm{buckling}}<R<R_{\mathrm{break-up}}\\ & \mathrm{if}\;\mathrm{ruptured}\;\mathrm{and}\;R\geq R_{\mathrm{rupture}} \end{array}$$

where *R*_buckling_ is the lower limit of the bubble radius, below which the lipid shell undergoes buckling, *R*_break-up_ is the upper limit of the bubble radius where the initial break-up of the shell occurs due to the decreased lipid concentration during bubble expansion. Between them, the lipid shell behaves elastically, where *χ* is the shell elasticity. Similar to previous studies [37,38,39,54,57], the *R*_break-up_ is assumed to be equal to the rupture radius *R*_rupture_ in this study. The *R*_buckling_ and *R*_rupture_ could be calculated by $R_{\mathrm{buckling}}=R_{0}/\sqrt{1+\sigma_{0}\left(R_{0}\right)/\chi }$ and $R_{\mathrm{rupture}}=R_{\mathrm{buckling}}/\sqrt{1+\sigma_{\mathrm{rupture}}/\chi }$ [24,32], where *R*_0_ is the initial bubble radius, $\sigma_{0}\left(R_{0}\right)$ is the initial surface tension at *R* = *R*_0_. The *σ*_rupture_ has been varied between 0.072 N/m for water and 1 N/m for different shells in the original work of Marmottant [24]. However, we assume $\sigma_{\mathrm{rupture}}=\sigma_{\mathrm{tissue}}$ in this paper referring to previous studies [36,37,38,39], where $\sigma_{\mathrm{tissue}}$ is the surface tension of the surrounding medium.

To take the shear-thinning behavior of the lipid-coated microbubbles into account, the widely used Cross law was adopted in the current work, and thereby the modification on the shell viscosity can be made as follows [32]:

(6) $$\kappa_{s}=\frac{\kappa_{0}}{1+\alpha \left|\dot{R}\right|/R}$$

where *κ*_0_ is the shell viscous parameter, *α* is a characteristic time constant for describing the characteristics of the lipid shell and the $\dot{R}/R$ can be treated as the shear rate of the shell.

### 2.4. Numerical Simulation Conditions

The pressure inside the *i*th microbubble *p_g,i_* is assumed to obey the van der Waals equation [53,57]:

(7) $$p_{g,i}=\left(p_{0}+\frac{2\sigma_{0}(R_{i0})}{R_{i0}}\right){\left(\frac{R_{i0}^{3}-h_{i}^{3}}{R_{i}^{3}-h_{i}^{3}}\right)}^{n}$$

where *p*_0_ was the atmospheric pressure, *σ*_0_*(R_i_*_0_) is the initial surface tension at *R* = *R_i_*_0_, *h_i_* = *R_i_*_0_/5.6 is the van der Waals hard-core radius for *i*th microbubble [58], *n* is the polytropic exponent of the gas within the microbubble.

According to pressure equilibrium at the bubble wall, the pressure in the surrounding medium at the wall of the *i*th microbubble was given by [8,26,57]:

(8) $$p_{s,i}=p_{g,i}-\frac{2\sigma \left(R\right)}{R_{i}}+{\tau_{rr}|}_{R_{i}}-\frac{4\kappa_{s}{\dot{R}}_{i}}{R_{i}^{2}}-p_{0}-p_{a}\left(t\right)$$

where $\tau_{rr}{|}_{R_{i}}$ is introduced to be able to couple the cavitation model with the viscoelastic model of the surrounding medium. The non-linearity of the ultrasound wave due to non-linear propagation, diffraction, or attenuation was not considered in this paper, and the acoustic pressure *p_a_*(*t*) = −*p_A_*sin(2π*ft*), where *p_A_* and *f* are the ultrasound amplitude and frequency, respectively.

Unless specified otherwise, the parameters were set as follows [32,38,55,59]: *f* = 1 MHz, *ρ* = 1050 kg/m^3^, *c* = 1540 m/s, *χ* = 0.44 N/m, *κ*_0_ = 5 × 10^−8^ kg/s, *α* = 3 μs, *σ*_0_ = 0.01 N/m, *σ*_tissue_ = 0.056 N/m, *n* = 1.07, *μ* = 15 mPa·s, *G* = 20 kPa, *λ*_1_ = 3 ns, *p*_0_ = 1.01 × 10^5^ Pa, *R*_10_ = 1 μm, *R*_20_ = 5 μm, *d*_1,2_ = *d*_2,1_ = 20 μm. The ordinary differential equations (ODEs) were solved numerically by using the ODE15s solver built in MATLAB (MathWorks Inc., R2018a, Natick, MA, USA) with a time step of 0.001/f, a relative tolerance of 10^−10^ and an absolute tolerance of 10^−11^.

## 3. Results

### 3.1. Dynamics of Two Interacting Encapsulated Microbubbles in the Viscoelastic Medium

**Figure 2:**
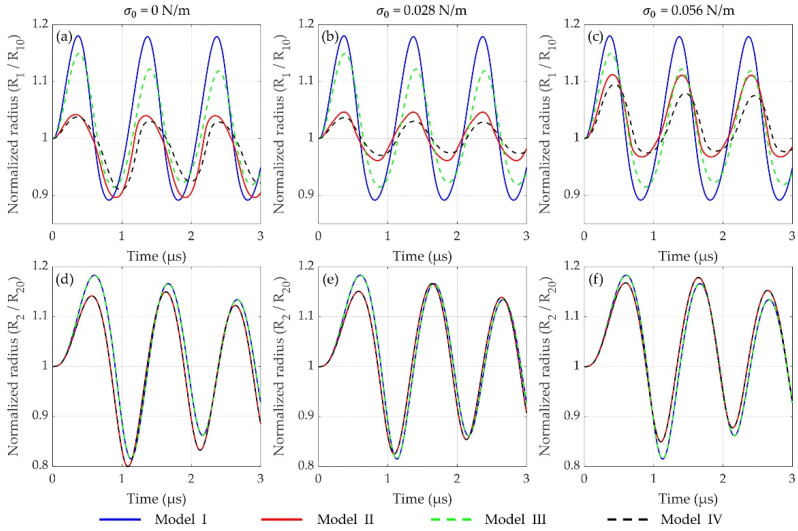
Bubble dynamics of two microbubbles (*R*_1_ and *R*_2_) in the viscoelastic tissue predicted by different models (I–IV) at different values of initial surface tension *σ*_0_ (*p_A_* = 100 kPa). (**a**–**c**) Normalized radii of a small bubble (*R*_1_) with an initial radius of *R*_10_ = 1 μm and (**d**–**f**) a large bubble (*R*_2_) with *R*_20_ = 5 μm as a function of time. Model I and model II represent uncoated and lipid-coated microbubbles without considering bubble–bubble interactions, respectively, whereas model III and model IV represent the corresponding cases with considering bubble–bubble interactions at *d_i,j_* = 20 μm.

For two lipid-shelled microbubbles having different initial radii (*R*_10_ = 1 μm and *R*_20_ = 5 μm) within a soft tissue, the bubble dynamics simulated by different models are first compared as shown in Figure 2. It exhibits a representation of the effects of encapsulating shell and inter-bubble interactions on the bubble dynamics at *p_A_* = 100 kPa. Model I and model II simulate two uncoated and two lipid-coated microbubbles oscillating in a soft tissue without considering bubble–bubble interactions, respectively, while model III and model IV represent the corresponding cases with considering bubble–bubble interactions. It can be seen that the oscillations of two microbubbles are prominently dampened by the inclusion of the lipid shells (model I versus model II, and model III versus model IV), and different behaviors are presented for different values of initial surface tension *σ*_0_, highlighting the significant effects of shell properties on bubble dynamics. For the effects of bubble–bubble interactions, it shows that the oscillations of the small bubble (*R*_1_) are distinctly suppressed (Figure 2a–c), whereas in those of the large one (*R*_2_), no obvious changes remain (Figure 2d–f), as compared with the cases without considering bubble–bubble interactions, respectively.

**Figure 3:**
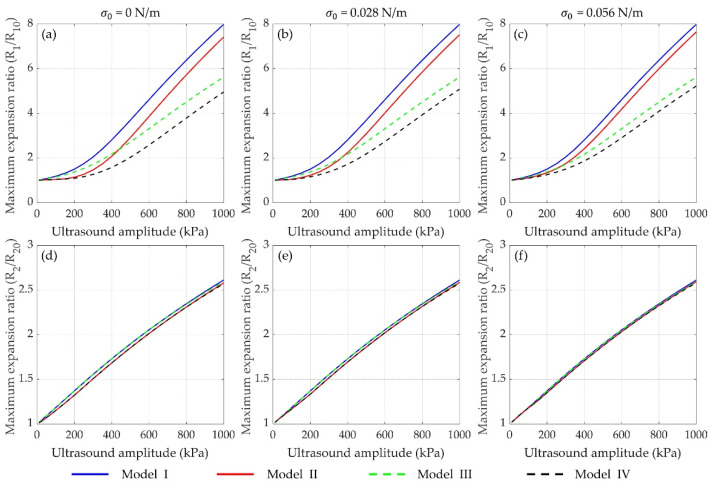
Maximum expansion ratios of (**a**–**c**) the small bubble (*R*_1_) and (**d**–**f**) the large bubble (*R*_2_) as function of ultrasound amplitude with the same conditions as Figure 2.

Furthermore, the maximum expansion ratios (*R*_max_/*R*_0_) of the two microbubbles as function of ultrasound amplitude *p_A_* are presented in Figure 3. It shows that the maximum expansion ratios of both microbubbles increase dramatically with the applied ultrasound amplitude increasing for the four models. As shown in Figure 3a–c, distinct suppression effects on the bubble oscillations are observed with considering the influences of the encapsulated shells and/or the bubble–bubble interactions, in accordance with Figure 2. Moreover, the difference of maximum expansion ratios for the small bubble between the models of I and III as well as the models of II and IV becomes larger and larger as the ultrasound amplitude increases, which indicates that a much stronger suppression effect exerts on the dynamics of the small bubble, probably due to the stronger bubble–bubble interactions at high acoustic pressures [48,57]. It is worth noting that the maximum expansion ratio is more than twice of the initial radius (*R*_max_/*R*_0_ ≥ 2) at high acoustic pressures, where the bubble often undergoes a short and violent collapse dominated by inertial forces, termed as inertial cavitation [14,15,16]. Additionally, it also demonstrates that both shell properties and bubble–bubble interactions exhibit significant effects on the small bubble rather than the large one. Therefore, in the following, we mainly focus on the dynamic behaviors and the inertial cavitation threshold of the small bubble, considering the bubble–bubble interactions with a large bubble.

### 3.2. Effects of Shell Properties on the Bubble Dynamics and Inertial Cavitation Threshold

#### 3.2.1. Effects of Initial Surface Tension

**Figure 4:**
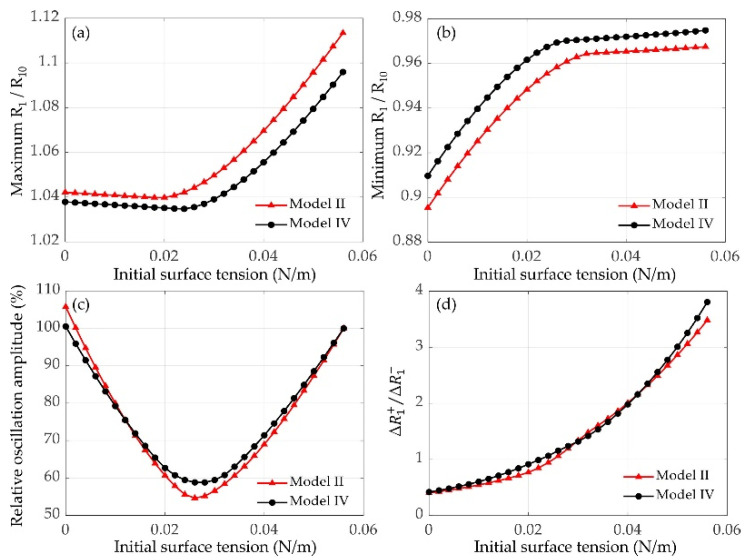
(**a**) Maximum and (**b**) minimum normalized radii of the small bubble (*R*_1_/*R*_10_) in two lipid-coated microbubbles without (model II) and with (model IV) bubble–bubble interactions as a function of initial surface tension *σ*_0_ (*P_A_* = 100 kPa, *d_i,j_* = 20 μm). (**c**) The relative oscillation amplitudes of lipid-coated bubbles with different *σ*_0_ to the uncoated bubble with *σ*_0_ = 0.056 N/m. (**d**) The ratios of the positive and negative radius excursions $\Delta R^{+}/\Delta R^{-}$ as a function of initial surface tension.

It has been shown in Figure 2 and Figure 3 that the initial surface tension *σ*_0_ of the lipid-coated microbubble has evident effects on its dynamic behaviors. The *σ*_0_ varies when using different manufacturing methods and it can also be altered by varying the ambient pressure in the surrounding medium [38,60,61]. Figure 4 exemplifies the effects of *σ*_0_ on the dynamic behaviors of two encapsulated microbubbles without/with considering bubble–bubble interactions. The maximum and minimum normalized radii of the small bubble (*R*_1_/*R*_10_) both increase with the *σ*_0_ increasing, as shown in Figure 4a,b, respectively. The relative oscillation amplitudes of the lipid-coated bubbles with different *σ*_0_ to the uncoated bubble (*σ*_0_ = 0.056 N/m) are also shown in Figure 4c. It indicates that the oscillation amplitude first decreases and then increases as the *σ*_0_ increases. Furthermore, the ratios of the positive and negative radius excursions $\Delta R^{+}/\Delta R^{-}$, defined by $\Delta R^{+}=\max\left(R_{1}\right)-R_{10}$ and $\Delta R^{-}=R_{10}-\min\left(R_{1}\right)$, are presented in Figure 4d. It can be observed that there is a compression-dominated behavior ($\Delta R^{+}/\Delta R^{-}<1$) for the microbubbles with smaller *σ*_0_ (*σ*_0_ < 0.022 N/m), whereas an expansion-dominated behavior ($\Delta R^{+}/\Delta R^{-}>1$) is observed for the microbubbles with larger *σ*_0_, which is consistent with previous studies on the dynamics of a single lipid-coated microbubble in water [37]. It can be explained that variations in *σ*_0_ changes the *R*_buckling_ and *R*_rupture_ which in turn change the dynamical behaviors of the bubbles.

#### 3.2.2. Effects of Shell Elasticity and Viscosity

**Figure 5:**
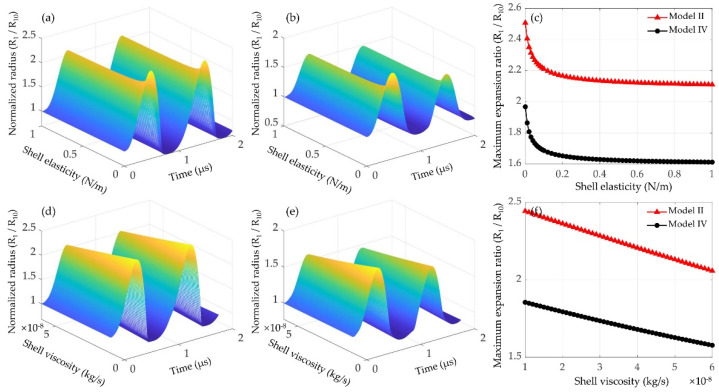
Normalized radii of a 1 μm lipid-coated bubble as a function of time (**a**) without (model II) and (**b**) with (model IV) considering the inter-bubble interactions with a large lipid-coated bubble (*R*_20_ = 5 μm), and (**c**) the corresponding maximum expansion ratios at different shell elasticities, while (**d**–**f**) represent those at different shell viscosities. The amplitude of applied ultrasound is 400 kPa, and the inter-bubble distance is 20 μm.

The effects of shell elasticity and viscosity on the dynamics of lipid-coated microbubbles without/with considering the bubble–bubble interactions were further examined. Shell property of commercial lipid-coated agents estimated through individual microbubble experiments has been summarized [62]. It shows that the shell elasticity and viscosity are varied prominently [62]. The range of simulation parameters examined in this paper were set to cover the main range of the variations. Under ultrasound excitation at 400 kPa, examples of normalized bubble radii as a function of time and the maximum expansion ratios for a 1 μm lipid-coated bubble with different shell elasticities (0.001~1 N/m) are shown in Figure 5a–c. With the shell elasticity increasing, the amplitude of bubble oscillation and the maximum expansion ratio are gradually decreased. A larger decrease rate can be observed for the microbubbles coated by the lipid shells with smaller elasticities. Moreover, the effects of shell viscosity on the bubble dynamics and the maximum expansion ratios are presented in Figure 5d–f. The shell viscosity is chosen as 1 × 10^−8^~6 × 10^−8^ kg/s and other conditions are the same as above. It is found that the bubble oscillations are distinctly reduced as the shell viscosity increases, consequently leading to a linear decrease in the maximum bubble expansion ratio. Figure 5 exemplifies that both the shell elasticity and viscosity would dramatically reduce the bubble oscillations, highlighting that the dynamics of encapsulated microbubbles might be controlled by modifying the shell elasticity and viscosity.

#### 3.2.3. Effects of Shell Properties on Inertial Cavitation Threshold

**Figure 6:**
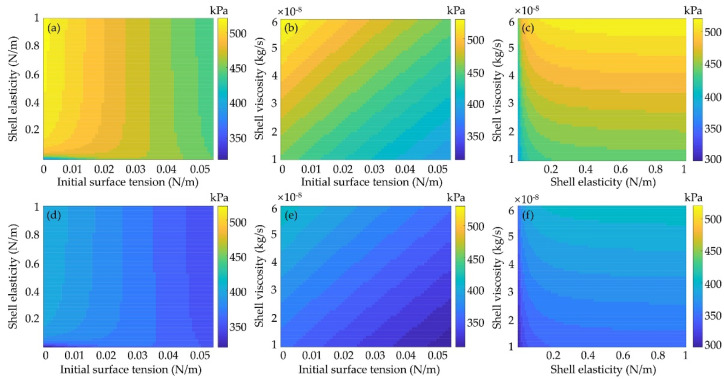
Effects of shell properties on the inertial cavitation threshold of a 1 μm lipid-coated microbubble (**a**–**c**) with and (**d**–**f**) without considering the inter-bubble interactions with a large lipid-coated bubble (*R*_20_ = 5 μm). Threshold mapping with (**a**,**d**) variation of the initial surface tension and the elasticity of shells, (**b**,**e**) variation of the initial surface tension and the viscosity of shells and (**c**,**f**) variation of the shell elasticity and the shell viscosity. The inter-bubble distance is 20 μm.

The inertial cavitation is determined by the bubble oscillations with *R*_max_/*R*_0_ = 2 as a criterion [14,15,16,17,18]. Therefore, the effects of shell properties, including the initial surface tension as well as the shell elasticity and viscosity, on the inertial cavitation threshold of a 1 μm lipid-coated microbubble with/without considering the inter-bubble interactions with a large lipid-coated bubble (*R*_20_ = 5 μm) were investigated. The mappings of inertial cavitation threshold at different shell properties with considering the bubble−bubble interactions are presented in Figure 6a–c. It can be seen that by increasing the initial surface tension, the inertial cavitation threshold decreases gradually, as shown in Figure 6a,b. With the shell viscoelasticity increasing, the inertial cavitation threshold increases, and the effect of the shell viscosity is much stronger than that of the shell elasticity, especially at larger shell elasticities (Figure 6c). Furthermore, compared with the inertial cavitation thresholds obtained without considering the bubble−bubble interactions (Figure 6d–f), it is obvious that the inertial cavitation thresholds are prominently higher as the bubble−bubble interactions were taken into account. Figure 6 exemplifies that the encapsulating shell of microbubble would increase its inertial cavitation threshold and illustrates the dependence between the inertial cavitation threshold and the initial surface tension, the elasticity and the viscosity of the encapsulating shell. This might serve as a potential strategy to reduce the inertial cavitation threshold with proper modifications of the shell properties for achieving safe and efficient ultrasound therapeutic applications.

### 3.3. Effects of Inter-Bubble Interactions on the Bubble Dynamics and Inertial Cavitation Threshold

#### 3.3.1. Effects of the Inter-Bubble Distance

**Figure 7:**
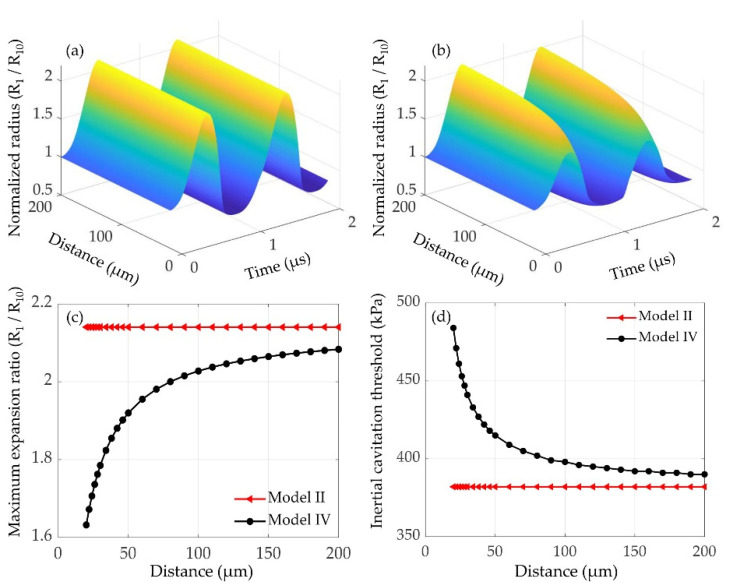
Effects of bubble−bubble interactions on the dynamics and inertial cavitation threshold of the small lipid-coated bubble (*R*_10_ = 1 μm) at different inter-bubble distances (*d_i,j_* = 20 μm−200 μm, *p_A_* = 400 kPa, *R*_20_ = 5 μm), including the normalized radii as a function of time (**a**) without (model II) and (**b**) with (model IV) considering the inter-bubble interactions, (**c**) the maxima of the normalized radii and (**d**) the inertial cavitation threshold as function of the inter-bubble distance.

As indicated by Equation (1), the oscillations of the interacting microbubbles would be severely affected by the inter-bubble distance. Thus, at different inter-bubble distances (20~200 μm), the normalized radius of a 1 μm lipid-coated microbubble as a function of time without and with considering the inter-bubble interactions with a large lipid-coated bubble (*R*_20_ = 5 μm) are shown in Figure 7a,b, respectively. The oscillations of the influenced bubble are significantly suppressed, and with the inter-bubble distance increasing, the difference between the corresponding maximum expansion ratios shown in Figure 7c becomes much smaller. Furthermore, as shown in Figure 7d, with the influence of the nearby bubble, the inertial cavitation threshold of the affected microbubble is noticeably higher than that of the isolated microbubble. Additionally, with the inter-bubble distance increasing, the difference of inertial cavitation threshold between the isolated and affected microbubbles becomes smaller due to the reduced inter-bubble interactions. Figure 7 indicates that increasing the inter-bubble distance could reduce the suppression effect on the bubble dynamics due to a decrease in the inter-bubble interactions, consequently leading to a distinct decrease in the inertial cavitation threshold.

#### 3.3.2. Effects of the Initial Radius of the Nearby Microbubble

**Figure 8:**
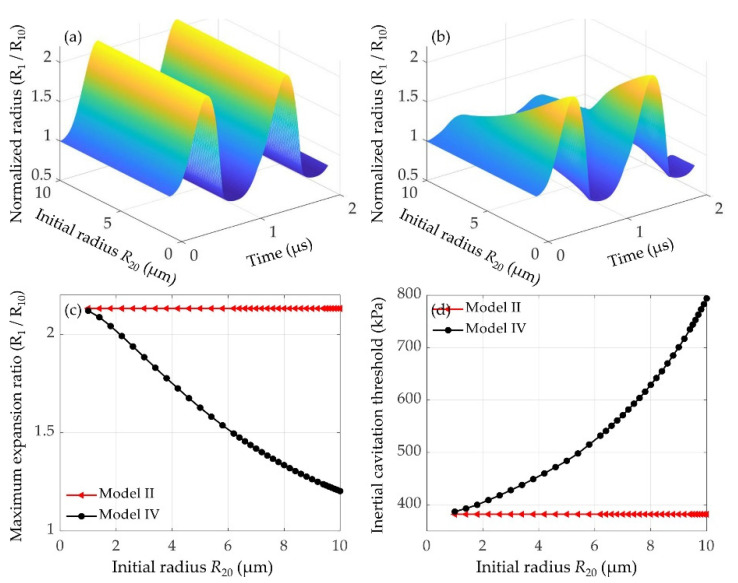
Dynamics and inertial cavitation threshold of a 1 μm lipid-coated bubble with the presence of a nearby microbubble having different initial radii (*R*_20_ = 1–10 μm) at *p_A_* = 400 kPa and *d_i,j_* = 20 μm, including the bubble dynamics as a function of time (**a**) without (model II) and (**b**) with (model IV) considering the bubble–bubble interactions, (**c**) the maxima of the normalized radii and (**d**) the inertial cavitation threshold as function of the initial radius of the nearby microbubble *R*_20_.

Considering that the size of microbubble might affect the cavitation dynamics, and then affect the oscillations of nearby microbubbles, therefore the effects of the initial radius of a large lipid-coated microbubble on the dynamics and inertial cavitation threshold of a 1 μm lipid-coated microbubble were examined. Normalized radius of the microbubble as a function of time without and with considering the inter-bubble interactions at different initial radii of the nearby microbubbles are shown in Figure 8a,b, respectively. Furthermore, the maximum expansion ratios in both cases are presented in Figure 8c. It shows that the oscillations and maximum expansion ratios of the isolated microbubble under all conditions are constant, whereas the bubble oscillations and maximum expansion ratios with considering the inter-bubble interactions decrease gradually as the initial radius of the nearby microbubble increases. Consequently, as shown in Figure 8d, the inertial cavitation threshold of the affected microbubble is higher than that of the isolated microbubble, and the difference of inertial cavitation threshold becomes much larger with the initial radius of the nearby bubble increasing. It can be explained that stronger suppression effects exert on the small bubble when the nearby microbubble has a larger bubble radius.

### 3.4. Effects of Medium Viscoelasticity on the Bubble Dynamics and Inertial Cavitation Threshold

**Figure 9:**
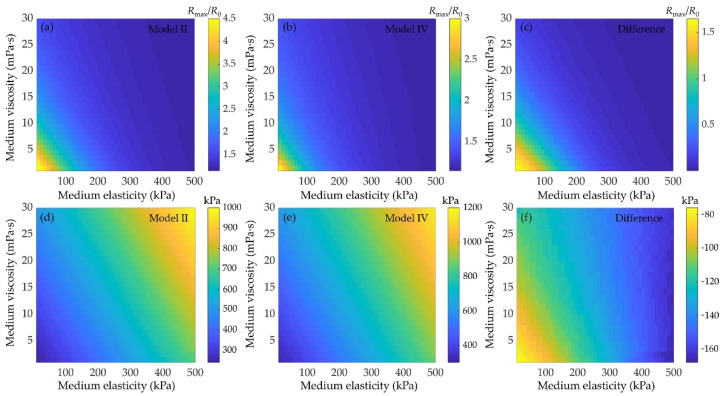
Effects of medium viscoelasticity on the bubble dynamics and inertial cavitation threshold. Maximum expansion ratio of a 1 μm lipid-coated microbubble at different elasticities and viscosities of the surrounding medium (**a**) without (model II) and (**b**) with (model IV) considering bubble−bubble interactions (*p_A_* = 400 kPa, *R*_10_ = 1 μm, *R*_20_ = 5 μm and *d_i,j_* = 20 μm), and (**c**) represents the difference between them. (**d**–**f**) represent the corresponding results of inertial cavitation threshold.

The effects of medium viscoelasticity on the bubble dynamics and inertial cavitation threshold are examined as shown in Figure 9. Examples of the maximum expansion ratio of a 1 μm lipid-coated microbubble without and with considering the bubble−bubble interactions at different medium elasticities (10~500 kPa) and viscosities (1~30 mPa·s) are shown in Figure 9a,b, respectively. Corresponding, the inertial cavitation thresholds of the microbubbles at different medium viscoelasticity are shown in Figure 9d,e, respectively. It showed similar variation tendencies in both cases, i.e., increasing the medium elasticity and viscosity, the bubble oscillations are noticeably suppressed, and in turn the inertial cavitation threshold increases, which agree well with the previous work [28,34,41,42,55,57]. Comparing the results obtained without and with considering the bubble−bubble interactions, it demonstrates that the difference of the maximum expansion ratios between the isolated and affected microbubbles becomes smaller (Figure 9c), but the difference of inertial cavitation threshold becomes larger (Figure 9f), with the medium viscosity and/or elasticity increasing. It indicates that increasing the medium viscoelasticity would suppress the oscillations of microbubble, which in turn reduce the bubble−bubble interactions, exactly as described in previous studies [55,57]. However, the increase in the medium viscoelasticity can also distinctly suppress the bubble oscillations, and result in the onset of inertial cavitation requiring higher acoustic pressure threshold, even if the suppression effect of the bubble−bubble interactions on the bubble oscillations has been reduced.

## 4. Discussion

Ultrasound in combination with encapsulated microbubbles is playing more and more roles in both diagnostic and therapeutic applications. It has been demonstrated that ultrasound-induced outcomes are primarily dependent on the bubble dynamics. However, the addition of an encapsulating shell and the viscoelasticity of the surrounding medium as well as the influences of nearby microbubbles would dramatically increase the complexity and difficulty in understanding the bubble dynamics. Here, we sought to comprehensively investigate the dynamic behaviors and inertial cavitation threshold of two coupled microbubbles with lipid shells via developing a comprehensive model with simultaneously considering the influences of the encapsulating shell, the surrounding medium and the nearby microbubbles. Furthermore, the effects of the shell properties, the bubble–bubble interactions and the viscoelasticity of the surrounding medium were analyzed.

For the effects of the encapsulating shells, the bubble dynamics and the inertial cavitation threshold of uncoated microbubbles and lipid-coated microbubbles are compared by utilizing a nonlinear model accounting for nonlinear changes of both shell viscosity and elasticity. The numerical results confirm that the encapsulating shell, which is the additional material present at the gas–liquid interface to stabilize microbubbles against dissolution and coalescence, would restrain the radial oscillations of microbubble as compared with the uncoated bubbles (Figure 2 and Figure 3). Moreover, in comparison with the linear viscoelastic model of the shell [15,28,54], the nonlinear model used in the present study shows a great advantage for describing the dynamic behaviors of the lipid-coated microbubbles whose shells behave in a nonlinear viscoelastic manner. For instance, the typical compression-only behavior of the lipid-coated microbubbles has been properly simulated by the model, where radial changes at the compression phase ($\Delta R^{-}=R_{0}-\min\left(R\right)$) are more than those at the expansion phase ($\Delta R^{+}=\max\left(R\right)-R_{0}$). This behavior can be attributed to the buckling and rupture of the lipid shell, which results in a radius-dependent shell elasticity as described by the Marmottant model shown in Equation (5). 

Furthermore, it also demonstrates that the magnitude of compression-only behavior depends on the initial surface tension *σ*_0_ of the lipid-coated microbubble. There seems to be two typical behaviors (Figure 4), showing the compression-dominated behavior as the *σ*_0_ is small, but it disappears, and the expansion-dominated behavior is observed when the *σ*_0_ increases. As indicated by Equation (5), the change of surface tension *σ*(*R*) with the bubble radius is one of the main factors of such special behaviors. Due to these asymmetric vibrations, the expansion ratio is larger and consequently the inertial cavitation threshold is lower for the bubble with a higher *σ*_0_ (Figure 4 and Figure 6) because of its expansion-dominated behavior. These results are in good agreement with the corresponding results for a single lipid-coated microbubble oscillating in water [37]. It can be explained that the microbubble with *σ*_0_ = 0 N/m is initially at the buckled state and has the largest rupture radius, whereas the microbubble with *σ*_0_ = 0.056 N/m is initially at the ruptured state and has the lowest buckling radius. Furthermore, the effects of shell properties, including the elasticity and dilatational viscosity, on the bubble dynamics and inertial cavitation threshold for the cases without and with considering the bubble–bubble interactions with a nearby bubble are compared (Figure 5 and Figure 6). The results indicate that either the shell elasticity or the shell viscosity would dampen the microbubble oscillations and in turn increase the inertial cavitation threshold in both cases. Understanding the relationships between the shell properties and the dynamic behaviors as well as the inertial cavitation thresholds of the encapsulated microbubbles is crucial for ultrasound imaging and therapeutic applications associated with the encapsulated microbubbles.

In addition to the effects of shell properties, it also shows that the interactions between the bubbles impose a distinct effect on the oscillations of the bubbles. Specifically, the dynamics of the smaller bubble could be significantly suppressed, whereas there is no obvious effect on the larger one, exhibiting a much stronger influence on the dynamics of the smaller bubble (Figure 1 and Figure 2). This is consistent with the previous results regarding the inter-bubble interactions of uncoated bubbles [48,53,55,57]. The suppression effects might be caused by the pressure radiated by each bubble at the location of the other bubble as shown by the last term in Equation (1). The influence of the radiated pressure may decrease the resultant pressure acting on the bubble and hence suppress the bubble oscillations [60]. Note that the larger the radiated pressure is, the stronger the suppression effect is, thus our results indicate that the pressure radiated by the larger bubble in the coupled two-bubble system is larger within the parameters examined in this paper. Moreover, for a coupled two-bubble system with different initial radii, the effects of bubble–bubble interactions on the radial oscillations and inertial cavitation threshold are also further examined via taking the small bubble as an example due to the stronger influences on the small bubble. Compared with the cases without considering bubble–bubble interactions, large differences in the bubble dynamics and inertial cavitation are noticeably observed. These differences could be attributed to the influence of the surrounding microbubbles, where the bubble–bubble interactions are likely to dampen the bubble oscillations and in turn increase the inertial cavitation threshold. With the distance between the bubbles increasing, the bubble–bubble interactions would be distinctly reduced (Figure 7). Additionally, the dynamics of interacting encapsulated microbubbles have been investigated in previous studies by assuming that all bubbles have the same initial radii for the sake of simplicity [31,46,54]. Nevertheless, in clinical and pre-clinical applications, bubbles are usually in polydisperse clusters despite the recent successful attempts in manufacturing mono-disperse bubbles [61,63]. To achieve a more accurate understanding on the dynamics of bubbles in realistic scenarios, the effects of the polydispersity of the microbubble size should be included. A few studies have investigated the interactions between encapsulated bubbles having different sizes in water, but with a primary focus on the secondary radiation force and the translational motions of the bubbles [45,52]. Thus, the effects of the size difference between the interacting encapsulated bubbles on the radial oscillations and the inertial cavitation threshold are further investigated in this paper. It indicates that with the initial radius of the nearby bubble increasing, the bubble oscillations are distinctly restrained and the inertial cavitation thresholds are prominently increased because of a stronger suppression effect on the small bubble, which is in agreement with the previous observations of uncoated bubbles [57].

Finally, it can be observed that with the elasticity and viscosity of the surrounding medium increasing, the bubble expansion ratios are much smaller, meaning that the viscoelasticity of the surrounding medium could significantly dampen the bubble oscillations and consequently increase the inertial cavitation threshold (Figure 9). It highlights the necessity to consider the influences of medium viscoelasticity while describing the acoustic cavitation in tissue fluid or blood that owns certain viscoelasticity. In addition, depending on the medium viscoelasticity, the effect of bubble–bubble interactions can occasionally become negligibly small as stated previously [55,57], hence there would be no obvious difference in the dynamics of bubbles that located in the media with sufficiently high elasticity or viscosity (or both) while comparing the two cases with/without considering the bubble−bubble interactions.

A better understanding of the physical interactions of lipid-coated microbubbles with ultrasound and nearby microbubbles in viscoelastic media would provide new insights and enable new approaches in both diagnostic and therapeutic ultrasound applications. Future studies need to investigate the dynamics of lipid-coated microbubbles via using this model within a wider range of parameters, such as at higher ultrasound frequency, which is of great importance for enhancing the ultrasound diagnostic imaging in clinic. Moreover, the mass transfer and rectified diffusion also need to be taken into account. They would influence the dynamics of the bubbles, and their effects can become significant in some therapeutic and engineering applications which employ long ultrasound pulses. Additionally, we only considered the inter-bubble interactions between two bubbles. However, in practical applications, bubbles are in polydisperse clusters and they interact with each other. For a more accurate understanding on the bubble dynamics, more bubbles should be considered in future studies. Therefore, the current should be extended to describe the clusters of more interacting lipid-encapsulated microbubbles. It may be easier to understand the dynamic behaviors and determine the inertial cavitation threshold since we exemplified the corresponding investigations with two interacting bubbles in the present study.

## 5. Conclusions

In this work, the cavitation dynamics and inertial cavitation threshold of lipid-coated microbubbles in viscoelastic media are comprehensively investigated with simultaneously considering the nonlinear viscoelastic behaviors of the shells, the bubble–bubble interactions and the viscoelasticity of the surrounding medium. The results suggest that the encapsulating shell, the inter-bubble interactions and the medium viscoelasticity would suppress bubble oscillations or expansion ratios of the bubbles. Due to the effects of the lipid shell, typical compression-only behavior or expansion-dominated oscillations due to buckling and rupture of the shell are observed. The closer the initial surface tension of the bubbles to the buckling stage or the ruptured stage is, the more prominent the compression-dominated or expansion-dominated behavior becomes, respectively. Consequently, the inertial cavitation threshold is noticeably reduced with the initial surface tension increasing. Nevertheless, increasing the shell elasticity and shell viscosity could dampen the expansion ratios of the bubbles and in turn increase the inertial cavitation threshold. Concerning the bubble–bubble interactions, it demonstrates that the larger bubble in the interacting two-bubble system tend to have a more significant influence on the smaller one, and the interactions grow stronger with a decrease in the distance between bubbles and/or an increase in the radius of the larger bubble, so that the bubble oscillations are restrained and the inertial cavitation threshold increases gradually. Moreover, with the elasticity or viscosity of the surrounding medium or both of them increasing, the inertial cavitation threshold significantly increases since the bubble oscillations are reduced dramatically under these conditions. This study may offer a better insight on the physical mechanisms of complex interactions of lipid-coated microbubble with ultrasound, nearby microbubbles and surrounding medium during ultrasound theranostic applications associated with encapsulated microbubbles.

## Data Availability

Not applicable.
